# Pleura – the new section in *Pleura and Peritoneum*

**DOI:** 10.1515/pp-2019-0016

**Published:** 2019-06-20

**Authors:** Maher Tabba

**Affiliations:** Pleura, Interventional Pulmonary Service, Tufts University School of Medicine, Boston, MA, USA

The *International Society for the Study of Pleura and Peritoneum* (ISSPP) was established last year with the aim to increase awareness on the organs of pleura, peritoneum, pericardium and other serosal surfaces in health and disease, including both benign and malignant pathologies. ISSPP also promotes research, innovation, education, training and patient care, as well as fostering interprofessional exchange.

As its title is suggesting, *Pleura and Peritoneum*, the official journal of ISSPP, is focusing on the organs “Pleura” and “Peritoneum”. However, so far, whereas the organ “Peritoneum” has been extensively discussed in this journal, articles on the organ “Pleura” are rather under-represented.

In order to change this, the journal’s Editors have decided to create a new section dedicated to the organ “Pleura”. This new section will promote basic research, education and excellence in understanding and managing pleural diseases, benign and malignant. It will also promote multidisciplinary approaches and foster the development and implementation of treatment guidelines for pleural diseases, which are largely missing.

Pleural diseases are a manifestation of a wide spectrum of medical conditions, ranging from simple illness such as pleurisy to life-threatening disease such as empyema or malignancy. It is estimated that more than one million patients develop pleural effusion annually, including adults and children. Multiple medical specialties are involved in the diagnosis and management of pleural diseases including pulmonology, thoracic surgery, thoracic medical oncology, thoracic radiation oncology, pulmonary pathology and thoracic radiology. Due the high incidence of pleural effusions, this knowledge should also be made available to internists and family practitioners, who are the primary medical care providers for a large number of patients. Increasing awareness of pleural diseases among a large segment of medical providers is very important to provide adequate care for the patients.

As the new Associate Editor responsible for the new section “Pleura” in this journal, I would like to invite medical specialists and researchers from all specialties above to participate to our effort by submitting their manuscripts to the journal. Together, we will setup a unique hub for scientists and a useful professional resource for clinicians. With the full support of the Editors of *Pleura and Peritoneum*, I am confident that we will be able to set a cornerstone for future advancement and development of scientific and medical knowledge on the organ “Pleura” in health and disease.

